# Fidaxomicin versus Vancomycin in the Treatment of* Clostridium difficile* Infection: Canadian Outcomes

**DOI:** 10.1155/2016/8048757

**Published:** 2016-05-24

**Authors:** Christine Lee, Thomas J. Louie, Karl Weiss, Louis Valiquette, Marvin Gerson, Wendy Arnott, Sherwood L. Gorbach

**Affiliations:** ^1^St Joseph's Healthcare, McMaster University, Hamilton, ON, Canada; ^2^Royal Jubilee Hospital, Island Health, Victoria, BC, Canada; ^3^Foothills Medical Centre, Calgary, AB, Canada; ^4^Maisonneuve-Rosemont Hospital, Montreal, QC, Canada; ^5^Université de Sherbrooke, Sherbrooke, QC, Canada; ^6^Humber River Hospital, Toronto, ON, Canada; ^7^Merck, Canada Inc., Kirkland, QC, Canada; ^8^Tufts University School of Medicine, Boston, MA, USA

## Abstract

*Background*. This analysis examined the efficacy of fidaxomicin versus vancomycin in 406 Canadian patients with* Clostridium difficile* infection (CDI), based on data from 2 randomized, clinical trials.* Methods*. Patients received fidaxomicin or vancomycin 1. Patients were assessed for clinical response recurrence of infection and sustained clinical response for 28 days after treatment completion. Patients at increased risk of recurrence were subjected to subgroup analyses.* Results*. Clinical response rates for fidaxomicin (90.0%) were noninferior to those with vancomycin (92.2%; 95% confidence interval for difference: −7.7, 3.5). However, fidaxomicin-treated patients had lower recurrence (14.4% versus 28.0%, *p* = 0.001) and higher sustained clinical response (77.1% versus 66.3%, *p* = 0.016). Compared with vancomycin, fidaxomicin was associated with lower recurrence rates in all subgroups, reaching statistical significance in patients with age ≥ 65 years (16.0% versus 30.9%, *p* = 0.026), concomitant antibiotic use (16.2% versus 38.7%, *p* = 0.036), and non-BI strains (11.8% versus 28.3%, *p* = 0.004). Higher sustained clinical response rates were observed for fidaxomicin compared with vancomycin in all subgroups; this was statistically significant in the non-BI subgroup (82.8% versus 69.1%, *p* = 0.021).* Conclusions*. In Canadian patients, fidaxomicin was superior to vancomycin in sustaining clinical response and reducing CDI recurrence.

## 1. Introduction

*Clostridium difficile* infection (CDI) is the leading cause of healthcare-associated infectious diarrhea, representing 15%–25% of diarrhea caused by antibiotics [1–3]. The Public Health Agency of Canada has collected national data on healthcare-associated CDI (HA-CDI) through the Canadian Nosocomial Infection Surveillance Program [4, 5]. Overall HA-CDI rates remained stable between 2009 and 2013 in hospitals participating in the Canadian Nosocomial Infection Surveillance Program; rates per 10,000 patient-days ranged from 5.36 to 6.65 [5]. HA-CDI rates vary by region, with the highest rates in Central and Western Canada and lowest rates in Eastern Canada, 6.23, 5.17, and 3.03 per 10,000 patient-days in 2013, respectively [5]. Overall CDI-attributable mortality rate (30 days after date of first positive CDI test) in adults was similar in 2009 (2.3%) and 2013 (3.1%) with a peak in 2011 (6.4%) [5].

The most dominant strain type isolated—representing approximately 40% of all isolates collected between 2007 and 2012—was the NAP1/BI/027 (BI) strain, which has been associated with increased toxin production and sporulation activity in vitro, infection severity, and patient mortality [4, 6–8]. The BI strain was more frequently isolated in Central Canada, with the proportion of BI isolates being almost double that observed in Western Canada (48.7% versus 27.0%, resp.; *p* ≤ 0.0001), and the BI strain was isolated from 16.7% of stool samples (*n* = 128) in the Eastern region [4].

Historical treatment options for CDI, vancomycin and metronidazole, are associated with clinical response rates of approximately 70% to 90% at the end of treatment. Based on previous evidence indicating that metronidazole is noninferior to vancomycin for the treatment of nonsevere CDI, metronidazole has been recommended for patients with mild-to-moderate CDI [1, 9–11]. Vancomycin, shown to be superior to metronidazole in patients with severe disease at baseline, is the recommended treatment choice for severe CDI [1, 9–11]. However, recent phase 3 trial data showing that metronidazole is inferior to vancomycin, regardless of baseline disease severity, have brought into question these recommendations [12]. Both metronidazole and vancomycin are associated with unacceptably high recurrence rates [13, 14]. CDI recurrence, due to infection with the same strain or infection with a different strain, has been documented in up to 28% of metronidazole-treated patients and 27% of vancomycin-treated patients [12, 14–18]. The risk of recurrence increases with each episode, and the risk of further recurrences in patients with recurrent CDI is 42% to 65% [19, 20]. Specific risk factors may predispose patients to recurrence, including advanced age, immunocompromised status, renal dysfunction, concomitant antibiotic use, and prior CDI [19, 21–26].

Fidaxomicin (DIFICID), approved in Canada in 2012 for treatment of CDI, is an orally administered, minimally absorbed, bactericidal macrocyclic antibiotic [27–30]. Fidaxomicin inhibits RNA synthesis by blocking formation of the RNA polymerase open promoter complex but at an earlier stage and a different site compared to rifamycin [31, 32]. Fidaxomicin is a narrow-spectrum antibiotic with a high degree of specificity against* C. difficile* [31, 33, 34]. Fidaxomicin is bactericidal against* C. difficile* in vitro with a minimum inhibitory concentration range of ≤0.001 to 1 *μ*g/mL, inhibits sporulation, and prevents future spore formation [35, 36]. In vitro studies show that fidaxomicin suppresses expression of the genes* tcdA* and* tcdB* and the regulatory gene* tcdR* and strongly inhibits the production of toxins A and B [37]. Furthermore, the administration of fidaxomicin for CDI has a minimal effect on the protective gut microbiota [38, 39].

Two phase 3, randomized, controlled double-blind trials conducted in the United States, Canada, and Europe showed that although fidaxomicin was noninferior to vancomycin at initial clinical response (end of treatment), the relative rate of recurrence was significantly less in fidaxomicin-treated patients (approximately half that observed in vancomycin-treated patients) [16, 17]. In addition, a significantly higher rate of sustained clinical response was observed in patients treated with fidaxomicin compared with those treated with vancomycin, based on follow-up through 28 ± 2 days after the end of treatment [16, 17]. This study comprises a post hoc analysis of Canadian patients from the 2 phase 3 clinical trials. Canadian trial sites contributed 37% of all modified intention-to-treat (mITT) patients in these registration trials, offering outcomes perspective based on relatively uniform care pathways in a public healthcare system. This study was conducted predominately to determine whether the outcomes for the subset of patients enrolled at Canadian sites were similar to the previously published overall trial outcomes [16] given the evidence that the hypervirulent strains such as NAP7 and NAP8 may be increasing in Canada [40]. The efficacy of fidaxomicin versus vancomycin is also assessed in clinical outcomes in patients with age ≥65 years, concomitant antibiotic use, cancer, renal dysfunction, and the BI strain versus non-BI strains.

## 2. Methods

### 2.1. Study Design

Two phase 3 multicenter, double-blind, randomized, noninferiority trials (OPT-80-003 and OPT-80-004) investigated the efficacy and safety of fidaxomicin versus vancomycin in the treatment of CDI [16, 17] (The two trials are registered with NCT00314951 and NCT00468728, http://www.clinicaltrials.gov). Study 003 recruited patients from 62 sites (US and Canada), and study 004 recruited patients from 86 sites (US, Canada, and 7 European countries). Patients were randomly assigned to receive blinded study drugs which were overencapsulated and identical in appearance: oral fidaxomicin 200 mg twice daily with intervening placebo or oral vancomycin 125 mg four times daily for 10 days. Patients were assessed daily during the 10-day treatment period for 2 days after the end of the treatment period and at least weekly during the 28-day follow-up period. Patients were assessed at an end-of-treatment visit for clinical response, and patients meeting criteria for clinical response were followed up for 28 days after the end of treatment and for a final trial assessment (36–40 days after randomization). Eligible patients' stool samples, collected within 48 hours of randomization, were tested for toxins A and B using the enzyme immunoassay (Meridian Bioscience, Inc., Cincinnati, Ohio) at study sites and the isolation of* C. difficile* and the susceptibility were performed as described [16]. Restriction endonuclease analysis [41, 42] was carried out by Edward Hines, Jr. (Veterans Affairs Hospital). Both trials followed the same protocol and were conducted in accordance with the principles of the Declaration of Helsinki and Good Clinical Practice. Both study protocols and amendments were approved by institutional review boards at all centres. Informed consent was obtained from all patients.

### 2.2. Patients

Eligible patients were 16 years of age or older, had CDI (defined as > 3 unformed bowel movements [UBMs] in the 24 hours before randomization), and had* C. difficile* toxin A or B (or both) in stool within 48 hours before randomization. Patients could have received up to 4 doses of vancomycin or metronidazole in the 24 hours preceding randomization. Patients were excluded if they had experienced > 1 previous episode of CDI in the 3 months before randomization, had received CDI-active antibiotics other than vancomycin or metronidazole, as described (e.g., oral bacitracin, fusidic acid, or rifaximin), presented with life-threatening or fulminant disease (e.g., toxic megacolon), or had known inflammatory bowel disease. No previous exposure to fidaxomicin was allowed.

### 2.3. Outcomes

The primary end point was clinical response, defined as the resolution of diarrhea (≤3 UBMs per day for 2 consecutive days) with no further requirement for CDI therapy, as assessed 2 days after the end of the 10-day blinded treatment course. Secondary efficacy end points were CDI recurrence and sustained clinical response, also called global cure. Recurrence was defined as the reappearance of > 3 UBMs in any 24-hour period with* C. difficile* toxin A or B (or both) detected and the need for CDI retreatment. Sustained clinical response was defined as clinical response without subsequent recurrence at the final trial assessment (36–40 days after randomization). Clinical failure was defined as the persistence of diarrhea, the need for additional CDI therapy, or both.

Treatment outcomes were also evaluated for patients with age ≥ 65 years, concomitant antibiotic use, cancer, renal dysfunction, or the BI strain versus non-BI strains. Patients receiving concomitant antibiotics were identified as described [43]; topical antibiotics, treatments for CDI (≤4 doses of vancomycin or metronidazole in the 24 hours preceding randomization), and antifungal and antiviral agents with no antibacterial activity were not considered concomitant antibiotics. Patients with solid tumors and/or hematologic malignancies were identified by system organ class and preferred term from active medical history entries of case report forms after coding by Medical Dictionary for Regulatory Activities version 10.0, by indications for concomitant medication entries, or by treatment-emergent adverse events. To identify patients with renal dysfunction, creatinine clearance was used as an estimate of glomerular filtration rate and was calculated using the Cockcroft-Gault equation from blood collected at baseline (before the first dose of study drug) and at the end of treatment, as described [44]. Patients were categorized based on the National Kidney Foundation Kidney Disease Outcomes Quality Initiative criteria: normal, ≥ 90 mL/min/1.73 m^2^; impaired, < 90 mL/min/1.73 m^2^.

### 2.4. Statistical Analyses

Modified intention-to-treat (mITT) and per-protocol populations were analyzed in terms of clinical response, recurrence and sustained clinical response based on treatment with fidaxomicin versus vancomycin. Two-sided 95% confidence intervals were calculated from the differences between treatment groups, and *χ* ^2^ statistic was used to determine significance. Any *p* value < 0.05 was considered significant. Statistical analysis was carried out using R statistical software (3.1.1). Efficacy analyses were carried out on data obtained from all patients in the mITT population, consisting of all randomized patients who met inclusion criteria for diarrhea (>3 UBMs in 24 hours), had a positive* C. difficile* toxin test at baseline, and received at least one dose of study drug. Patients in the per-protocol population met the criteria for the mITT population and took at least 3 days of study drug for failures or 8 days for cures, had no major protocol violations, and had an end-of-therapy assessment for cure.

## 3. Results

**Figure 1:**
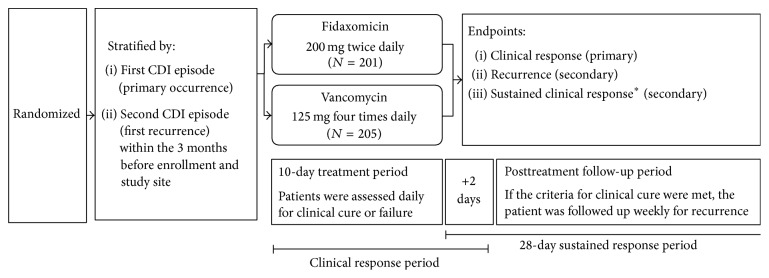
Study design. ^*∗*^Sustained clinical response was defined as clinical cure without subsequent recurrence at the final trial assessment (36–40 days after randomization).

A total of 406 patients were enrolled at Canadian sites and underwent randomization; 201 patients received fidaxomicin and 205 patients received vancomycin (Figure 1). Baseline characteristics were similar between treatment groups (Table 1). Most patients were female (62.1%), 45.6% were aged ≥ 65 years, and 52.2% (212/406) were inpatients. Using the described criteria [16], 163 patients (40.1%) had severe disease at baseline. Eighty patients (19.7%) received concomitant antibiotics and 13.3% had cancer (solid tumor, hematological malignancy, or both). Of the 342 patients (84.2%) with isolates available for restriction endonuclease analysis typing, 133 (38.9%) were infected with the BI strain (Tables 1 and 2). The BI strain was more common in Quebec (48.3%) and Ontario (58.9%) than in Western Canada (14.3%) and was more frequently isolated from inpatients (62.9%) than from outpatients (15.1%).

**Figure 2:**
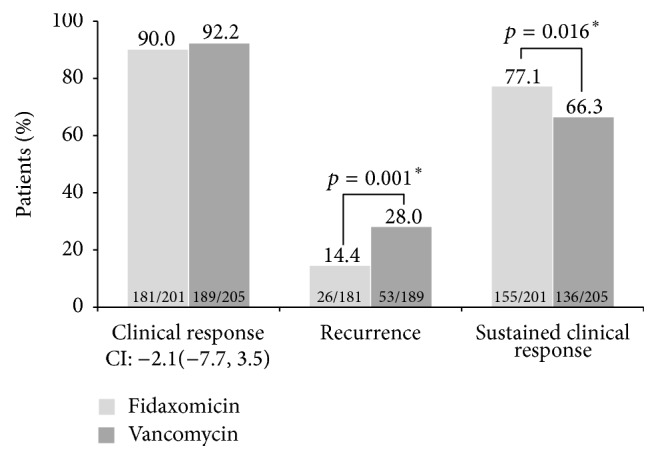
Rates of clinical outcomes within the modified intent-to-treat population. ^*∗*^ *p* < 0.05.

**Figure 3:**
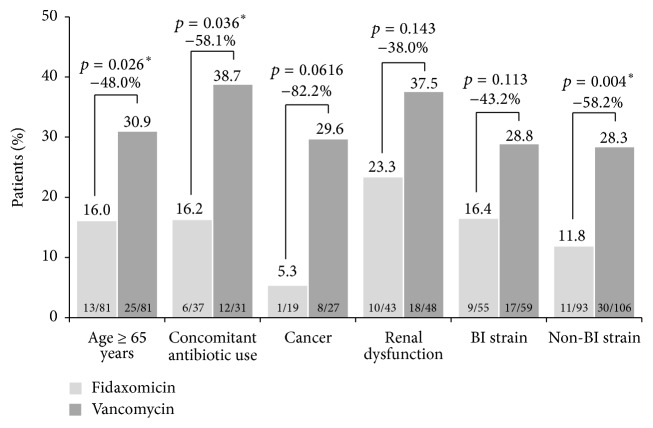
Rates of recurrence in subgroups. ^*∗*^ *p* < 0.05.

Overall outcomes in the Canadian population are shown (Figure 2). Clinical response rates were similar between arms (90.0% for fidaxomicin and 92.2% for vancomycin; 95% confidence interval for difference: −7.7, 3.5). Fidaxomicin was associated with a lower rate of recurrence compared with vancomycin (14.4% versus 28.0%, *p* = 0.001). At the end-of-study visit, 77.1% of patients in the fidaxomicin group and 66.3% of patients in the vancomycin group were considered to have met the criteria for sustained clinical response. This represented a 16.2% increase in sustained clinical response with fidaxomicin versus vancomycin, which was statistically significant (*p* = 0.016). Clinical response rates were similar between arms within all of the subgroups analyzed: age ≥ 65 years, concomitant antibiotic use, cancer, renal dysfunction, and BI strain (Table 3). Recurrence rates were lower for fidaxomicin than for vancomycin across all subgroups, and this difference was significant for subgroups with age ≥ 65 years, concomitant antibiotic use, and non-BI strains (Figure 3). Higher sustained clinical response rates were observed for fidaxomicin compared with vancomycin in all subgroup analyses, which were statistically significant in the non-BI subgroup (82.8% versus 69.1%, *p* = 0.021).

## 4. Discussion

Results demonstrated noninferiority of fidaxomicin compared with vancomycin for clinical response in the Canadian population of the 2 phase 3 trials. Treatment with fidaxomicin was associated with a lower recurrence rate within 4 weeks of completion of therapy and a higher sustained clinical response rate. This post hoc analysis comprised 406 Canadian patients, a subset of overall patients in the 2 phase 3 trials. This study was not powered to show statistical significance in this subset of patients or further subgroups of these Canadian patients. Nevertheless, these results agree with those reported for the international population of the 2 phase 3 trials.

As previously reported, 88.2% and 87.7% of patients treated with fidaxomicin and 86.8% and 85.8% of patients treated with vancomycin achieved clinical response in the phase 3 003 and 004 trials, respectively [16, 17]. The present analysis of the Canadian subset of these trials showed similar results, with 90.0% of fidaxomicin-treated patients and 92.2% of vancomycin-treated patients achieving clinical response. Clinical and statistical superiority of fidaxomicin over vancomycin for recurrence and sustained clinical response was also in agreement with the 003 and 004 trials data.

Treatment with concomitant antibiotics has been shown to compromise initial response to CDI therapy and durability of response [43]. According to the analysis of this subgroup, patients treated with fidaxomicin were significantly less likely to experience a recurrence than the vancomycin recipients. These results indicate that fidaxomicin is an appropriate therapy for patients with CDI receiving concurrent antibiotic(s). Fidaxomicin also significantly reduced recurrence in patients with ≥ 65 years of age, a population shown to be at risk for recurrent CDI, compared with vancomycin [24, 25]. There was a statistically significant, superior, sustained clinical response and reduction in recurrence rate in patients treated with fidaxomicin versus vancomycin in the non-BI subgroup, which is consistent with the phase 3 trials. There was also a trend toward reduction in recurrence in Canadian patients infected with the BI strain who received fidaxomicin compared with vancomycin. This was similar to the 004 trial, where recurrence rates were 22.2% and 38.0% for patients infected with the BI strain treated with fidaxomicin and vancomycin, respectively [17].

The key strengths of this analysis were its reliance on multicentre, double-blind, randomized trials, its consideration of subgroups at high risk of recurrence, and separate examination of the BI strain versus non-BI strains. Although the subgroups were large enough for meaningful clinical comparisons between fidaxomicin and vancomycin, these results should be interpreted with caution because the study was not powered to detect statistical differences within these subgroups.

Fidaxomicin is an effective treatment option for patients with CDI, and the greatest benefit is expected among those with known risk factors for recurrence. Compared with vancomycin, fidaxomicin reduces recurrence and increases sustained clinical response. Findings among Canadian patients were similar to overall international results, except the recurrence benefit observed in the BI subgroup of Canadian patients, where a greater reduction in recurrence was observed.

## Figures and Tables

**Table 1:** Patient and disease characteristics within the mITT population.

Characteristic	Fidaxomicin *n* = 201	Vancomycin *n* = 205	Total *n* = 406
Female, number (%)	127 (63.2)	125 (61.0)	252 (62.1)
UBMs per day, mean (SD)	8.1 (4.2)	7.8 (4.5)	8.0 (4.3)
Inpatient, number (%)	107 (53.2)	105 (51.2)	212 (52.2)
CDI antibiotics within previous 24 hours, number (%)	59 (29.4)	63 (30.7)	122 (30.0)
Previous CDI episode, number (%)	39 (19.4)	37 (18.0)	76 (18.7)
Severe CDI,^*∗*^ number (%)	83 (41.3)	80 (39.0)	163 (40.1)
Age ≥ 65 years, number (%)	93 (46.3)	92 (44.9)	185 (45.6)
Antibiotic use^†^, number (%)	43 (21.4)	37 (18.0)	80 (19.7)
Cancer, number (%)	23 (11.4)	31 (15.1)	54 (13.3)
Renal dysfunction^‡^, number (%)	119 (59.2)	107 (52.2)	226 (55.7)
BI strain^§^, number (%)	66 (40.0)	67 (37.9)	133 (38.9)

^*∗*^Severe CDI at baseline: ≥10 UBMs per day or white blood cell count > 15,000/mm^3^.

^†^Other than CDI treatment, during either CDI treatment or follow-up period.

^‡^Renal impairment at baseline: creatinine clearance < 90 mL/min/1.73 m^2^.

^§^Percentages are calculated from all patients and not from the 342 patients for whom strain type was available.

BI, BI/NAP1/027; CDI, *Clostridium difficile* infection; mITT, modified intention-to-treat; SD, standard deviation; UBMs, unformed bowel movements.

**Table 2:** Distribution of patients with BI and non-BI strains by region and hospitalization status at time of randomization.

	BI strain *n* = 133	Non-BI strain *n* = 209	Total *n* = 342
Region			
Quebec, number (%)	84 (48.3)	90 (51.7)	174
Ontario, number (%)	33 (58.9)	23 (41.1)	56
West (BC, AB, and SK), number (%)	16 (14.3)	96 (85.7)	112
Hospitalization status			
Inpatient, number (%)	107 (62.9)	63 (37.1)	170
Outpatient, number (%)	26 (15.1)	146 (84.9)	172

AB, Alberta; BC, British Columbia; BI, BI/NAP1/027; SK, Saskatchewan.

**Table 3:** Clinical response and sustained clinical response of subgroups within the modified intent-to-treat population.

Clinical response	Fidaxomicin number/number (%)	Vancomycin number/number (%)	Change (%)	Difference (CI)
Age ≥ 65 years	81/93 (87.1)	81/92 (88.0)	−1.1	−0.9 (−10.5, 8.7)
Concomitant antibiotic use	24/27 (88.9)	21/27 (77.8)	14.3	11.1 (−9.3, 30.0)
Cancer	19/23 (82.6)	27/31 (87.1)	−5.2	−4.5 (−24.4, 14.7)
Renal dysfunction	43/56 (76.8)	48/55 (87.3)	−12.0	−10.5 (−24.2, 4.0)
BI strain	55/66 (83.3)	59/67 (88.1)	−5.4	−4.7 (−16.6, 7.4)
Non-BI strain	93/99 (93.9)	106/110 (96.4)	−2.5	−2.4 (−8.7, 3.8)

Sustained clinical response	Fidaxomicin number/number (%)	Vancomycin number/number (%)	Change (%)	*p* value

Age ≥ 65 years	68/93 (73.1)	56/92 (60.9)	20.1	0.076
Concomitant antibiotic use	31/43 (72.1)	19/37 (51.4)	40.4	0.056
Cancer	18/23 (78.3)	19/31 (61.3)	27.7	0.184
Renal dysfunction	33/56 (58.9)	30/55 (54.5)	8.0	0.641
BI strain	46/66 (69.7)	42/67 (62.7)	11.2	0.393
Non-BI strain	82/99 (82.8)	76/110 (69.1)	19.9	0.021^*∗*^

BI, BI/NAP1/027; CI, confidence interval.

^*∗*^ *p* < 0.05.

## References

[1] Cohen S. H., Gerding D. N., Johnson S. (2010). Clinical practice guidelines for Clostridium difficile infection in adults: 2010 update by the Society for Healthcare Epidemiology of America (SHEA) and the Infectious Diseases Society of America (IDSA). *Infection Control and Hospital Epidemiology*.

[2] Dubberke E. (2012). *Clostridium difficile* infection: the scope of the problem. *Journal of Hospital Medicine*.

[3] Kim J. H., Muder R. R. (2011). *Clostridium difficile* enteritis: a review and pooled analysis of the cases. *Anaerobe*.

[4] Public Health Agency of Canada (2014). *Healthcare-Associated Clostridium Difficile Infections in Canadian Acute-Care hospitals: Surveillance Report January 1, 2007 to December 31, 2012*.

[5] Public Health Agency of Canada (2014). *Antimicrobial Resistant Organisms (ARO) Surveillance in Canadian Hospitals 2007–2012*.

[6] Miller M., Gravel D., Mulvey M. (2010). Health care-associated *Clostridium difficile* infection in Canada: patient age and infecting strain type are highly predictive of severe outcome and mortality. *Clinical Infectious Diseases*.

[7] Merrigan M., Venugopal A., Mallozzi M. (2010). Human hypervirulent *Clostridium difficile* strains exhibit increased sporulation as well as robust toxin production. *Journal of Bacteriology*.

[8] Warny M., Pepin J., Fang A. (2005). Toxin production by an emerging strain of *Clostridium difficil*e associated with outbreaks of severe disease in North America and Europe. *The Lancet*.

[9] Debast S. B., Bauer M. P., Kuijper E. J. (2014). European society of clinical microbiology and infectious diseases: update of the treatment guidance document for *Clostridium difficile* infection. *Clinical Microbiology and Infection*.

[10] Surawicz C. M., Brandt L. J., Binion D. G. (2013). Guidelines for diagnosis, treatment, and prevention of *Clostridium difficile* infections. *The American Journal of Gastroenterology*.

[11] Zar F. A., Bakkanagari S. R., Moorthi K. M. L. S. T., Davis M. B. (2007). A comparison of vancomycin and metronidazole for the treatment of *Clostridium difficile*–associated diarrhea, stratified by disease severity. *Clinical Infectious Diseases*.

[12] Johnson S., Louie T. J., Gerding D. N. (2014). Vancomycin, metronidazole, or tolevamer for *Clostridium difficile* infection: results from two multinational, randomized, controlled trials. *Clinical Infectious Diseases*.

[13] Johnson S., Gerding D., Davidson D. Efficacy and safety of oral vancomycin versus oral metronidazole for treatment of *Clostridium difficile* associated diarrhea.

[14] Vardakas K. Z., Polyzos K. A., Patouni K., Rafailidis P. I., Samonis G., Falagas M. E. (2012). Treatment failure and recurrence of *Clostridium difficile* infection following treatment with vancomycin or metronidazole: a systematic review of the evidence. *International Journal of Antimicrobial Agents*.

[15] Musher D. M., Aslam S., Logan N. (2005). Relatively poor outcome after treatment of *Clostridium difficile* colitis with metronidazole. *Clinical Infectious Diseases*.

[16] Louie T. J., Miller M. A., Mullane K. M. (2011). Fidaxomicin versus vancomycin for *Clostridium difficile* infection. *The New England Journal of Medicine*.

[17] Cornely O. A., Crook D. W., Esposito R. (2012). Fidaxomicin versus vancomycin for infection with *Clostridium difficile* in Europe, Canada, and the USA: a double-blind, non-inferiority, randomised controlled trial. *The Lancet Infectious Diseases*.

[18] Cornely O. A., Miller M. A., Louie T. J., Crook D. W., Gorbach S. L. (2012). Treatment of first recurrence of *Clostridium difficile* infection: fidaxomicin versus vancomycin. *Clinical Infectious Diseases*.

[19] McFarland L. V., Surawicz C. M., Greenberg R. N. (1994). A randomized placebo-controlled trial of *Saccharomyces boulardii* in combination with standard antibiotics for *Clostridium difficile* disease. *The Journal of the American Medical Association*.

[20] McFarland L. V. (1999). Recurrent *Clostridium difficile* disease: epidemiology and clinical characteristics. *Infection Control and Hospital Epidemiology*.

[21] D'Agostino R. B., Collins S. H., Pencina K. M., Kean Y., Gorbach S. (2014). Risk estimation for recurrent *Clostridium difficile* infection based on clinical factors. *Clinical Infectious Diseases*.

[22] Do A. N., Fridkin S. K., Yechouron A. (1998). Risk factors for early recurrent *Clostridium difficile*–associated diarrhea. *Clinical Infectious Diseases*.

[23] Kyne L., Warny M., Qamar A., Kelly C. P. (2001). Association between antibody response to toxin A and protection against recurrent *Clostridium difficile* diarrhoea. *The Lancet*.

[24] Garey K. W., Sethi S., Yadav Y., DuPont H. L. (2008). Meta-analysis to assess risk factors for recurrent *Clostridium difficile* infection. *Journal of Hospital Infection*.

[25] Eyre D. W., Walker A. S., Wyllie D. (2012). Predictors of first recurrence of *Clostridium difficile* infection: implications for initial management. *Clinical Infectious Diseases*.

[26] Abou Chakra C. N., Pepin J., Sirard S., Valiquette L. (2014). Risk factors for recurrence, complications and mortality in *Clostridium difficile* infection: a systematic review. *PLoS ONE*.

[27] Shue Y. K., Sears P. S., Shangle S. (2008). Safety, tolerance, and pharmacokinetic studies of OPT-80 in healthy volunteers following single and multiple oral doses. *Antimicrobial Agents and Chemotherapy*.

[28] Louie T., Miller M., Donskey C., Mullane K., Goldstein E. J. C. (2009). Clinical outcomes, safety, and pharmacokinetics of OPT-80 in a phase 2 trial with patients with *Clostridium difficile* infection. *Antimicrobial Agents and Chemotherapy*.

[29] Babakhani F., Gomez A., Robert N., Sears P. (2011). Killing kinetics of fidaxomicin and its major metabolite, OP-1118, against *Clostridium difficile*. *Journal of Medical Microbiology*.

[30] Sears P., Crook D. W., Louie T. J., Miller M. A., Weiss K. (2012). Fidaxomicin attains high fecal concentrations with minimal plasma concentrations following oral administration in patients with *Clostridium difficile* infection. *Clinical Infectious Diseases*.

[31] Artsimovitch I., Seddon J., Sears P. (2012). Fidaxomicin is an inhibitor of the initiation of bacterial RNA synthesis. *Clinical Infectious Diseases*.

[32] Babakhani F., Seddon J., Sears P. (2014). Comparative microbiological studies of transcription inhibitors fidaxomicin and the rifamycins in *Clostridium difficile*. *Antimicrobial Agents and Chemotherapy*.

[33] Credito K. L., Appelbaum P. C. (2004). Activity of OPT-80, a novel macrocycle, compared with those of eight other agents against selected anaerobic species. *Antimicrobial Agents and Chemotherapy*.

[34] Finegold S. M., Molitoris D., Vaisanen M.-L., Song Y., Liu C., Bolaños M. (2004). In vitro activities of OPT-80 and comparator drugs against intestinal bacteria. *Antimicrobial Agents and Chemotherapy*.

[35] Babakhani F., Bouillaut L., Gomez A., Sears P., Nguyen L., Sonenshein A. L. (2012). Fidaxomicin inhibits spore production in *Clostridium difficile*. *Clinical Infectious Diseases*.

[36] Goldstein E. J. C., Babakhani F., Citron D. M. (2012). Antimicrobial activities of fidaxomicin. *Clinical Infectious Diseases*.

[37] Babakhani F., Bouillaut L., Sears P., Sims C., Gomez A., Sonenshein A. L. (2013). Fidaxomicin inhibits toxin production in *Clostridium difficile*. *Journal of Antimicrobial Chemotherapy*.

[38] Tannock G. W., Munro K., Taylor C. (2010). A new macrocyclic antibiotic, fidaxomicin (OPT-80), causes less alteration to the bowel microbiota of *Clostridium difficile*-infected patients than does vancomycin. *Microbiology*.

[39] Louie T. J., Cannon K., Byrne B. (2012). Fidaxomicin preserves the intestinal microbiome during and after treatment of *Clostridium difficile* infection (CDI) and reduces both toxin reexpression and recurrence of CDI. *Clinical Infectious Diseases*.

[40] Mulvey M. R., Boyd D. A., Gravel D. (2010). Hypervirulent *Clostridium difficile* strains in hospitalized patients, Canada. *Emerging Infectious Diseases*.

[41] Clabots C. R., Johnson S., Bettin K. M. (1993). Development of a rapid and efficient restriction endonuclease analysis typing system for *Clostridium difficile* and correlation with other typing systems. *Journal of Clinical Microbiology*.

[42] Killgore G., Thompson A., Johnson S. (2008). Comparison of seven techniques for typing international epidemic strains of *Clostridium difficile*: restriction endonuclease analysis, pulsed-field gel electrophoresis, PCR-ribotyping, multilocus sequence typing, multilocus variable-number tandem-repeat analysis, amplified fragment length polymorphism, and surface layer protein a gene sequence typing. *Journal of Clinical Microbiology*.

[43] Mullane K. M., Miller M. A., Weiss K. (2011). Efficacy of fidaxomicin versus vancomycin as therapy for *Clostridium difficile* infection in individuals taking concomitant antibiotics for other concurrent infections. *Clinical Infectious Diseases*.

[44] Mullane K. M., Cornely O. A., Crook D. W. (2013). Renal impairment and clinical outcomes of *Clostridium difficile* infection in two randomized trials. *American Journal of Nephrology*.

